# Assessing veterinary professionals’ perspectives on community knowledge, attitudes, and practices regarding dog rabies in Turkana, Kenya

**DOI:** 10.3389/fvets.2025.1526551

**Published:** 2025-03-13

**Authors:** Moumita Das, María Sol Pérez Aguirreburualde, Job Ronoh Kipkemoi, Erenius Lochede Nakadio, Andres M. Perez, Melinda Wilkins

**Affiliations:** ^1^Center for Animal Health and Food Safety, College of Veterinary Medicine, University of Minnesota, St. Paul, MN, United States; ^2^Department of Veterinary Population Medicine, University of Minnesota, St. Paul, MN, United States; ^3^Turkana County Government Directorate of Veterinary Services, Lodwar, Kenya

**Keywords:** rabies, dogs, vaccination, knowledge, attitudes, practices, Kenya

## Abstract

In Kenya, rabies is a deadly zoonotic illness that has been recognized for over a century. The main reservoir and vector for human transmission of the disease is domestic dogs. Utilizing a Rabies Workshop in Turkana County, Kenya in November 2023, this study aimed to assess the knowledge, attitudes, and practices (KAP) of the local community people regarding rabies. Data were gathered using an online survey from a range of veterinary professionals, including public and private veterinarians, para-veterinarians, and community disease reporters, using a cross-sectional approach. Each participant acted as a representative respondent for the local communities in which they served. A one-way analysis of variance (ANOVA) was used to analyze occupational differences, while a two-sample *t*-test was conducted to evaluate regional differences. The results indicated that 42.4% of experts believed less than half of the community was knowledgeable about rabies, while 75.8% thought less than half could recognize the clinical signs in dogs. Likewise, the level of knowledge, positive attitudes, and practices regarding dog vaccination in Turkana was similarly less than 50%. Dogs were largely utilized for the purpose of security and guarding, and predominantly free to roam. The primary obstacles to dog vaccination encompassed insufficient awareness regarding rabies, a lack of information concerning immunization campaigns and the cost of vaccination. No statistical significance was found in the participant’s responses against their service locations, and their professions, except the positive attitudes toward veterinary care for dogs in different sub-counties (*p*-value = 0.03). The study indicates that utilizing any and/or all professionals can contribute valid surveillance data for rabies control efforts in Turkana. Overall, the rabies-related knowledge, attitudes, and practices among the Turkana community are unsatisfactory across all sub-counties. These findings have significant influence on policy development and decision-making process, highlighting the importance of targeted interventions to improve rabies awareness and vaccination rates in similar settings.

## 1 Introduction

Rabies has been a significant public health threat in Kenya for almost a century. The disease was initially discovered in a domestic dog in Nairobi in 1912, and in 1928, Lake Victoria witnessed the first recorded human case (1). Since then, the disease has affected almost every part of the country as it has expanded nationwide. Kenya employs a passive surveillance system, relying on reported rabies cases by health facilities, veterinary clinics, or the public rather than proactively identifying cases through systematic data collection or field investigations (2). An estimated 2000 people die from rabies each year, making it a significant danger for the country (3). The government has implemented several measures to combat this disease, including a National Rabies Elimination Strategic Plan (NRESP) to eliminate human rabies caused by dogs by 2030 (4). The NRESP includes mass dog vaccination nationwide. However, the implementation of these initiatives has faced significant challenges. In 2013, political devolution transitioned decision-making power regarding the distribution of national resources, opportunities for employment, medical services, transportation, and public works from the central Government to the 47 semi-autonomous counties (5). This administrative change complicated rabies vaccine procurement due to political influences and the varying priorities of local county governments. Furthermore, the privatization of veterinary services made the rabies vaccine expensive, previously it was supplied by the Central Government free of cost. These political and administrative changes led to a significant decrease in the dog immunization rate in recent years (6), making the NRESP very challenging to implement.

Knowledge, attitudes, and practice (KAP) studies focusing on canine rabies are widely conducted as a significant resource for controlling the disease worldwide. A study in Indonesia found substantial gaps in rabies knowledge, highlighting the need for community education and awareness programs (7). Similarly, research in Zimbabwe underscored the importance of understanding health-seeking behavior and the vaccination of dogs against rabies (8). Another study in Chad emphasized the need for improving inter-sectoral collaboration between human and animal health science (9). Similar calls for collaboration have been made in Kenya and Ethiopia, advocating for efforts to control free-roaming dogs, raise awareness, and expand vaccination campaigns (10).

The present investigation was carried out in Turkana county, located in the northwestern region of Kenya. Turkana County’s geographical area spans roughly 77,000 square kilometers, making it the second-largest county in the country (11). The region spans from the western shores of Lake Turkana, the largest desert lake in the world, to the Great African Rift Valley. It reaches across the borders of Uganda on the west, South Sudan to the northwest, Ethiopia to the north, and the Cherangani Hills to the south. This region in Kenya is classified as an arid and semi-arid land (ASAL), historically inhabited by pastoralists. Livestock husbandry accounts for around 62% of the primary income of the local population, while agro-pastoralism contributes 20%, fishing contributes 12%, and temporary employment provides 8% of revenue (12).

The distinctive landscape of Turkana, combined with its firm veterinary services, especially in surveillance and coordination of Community Animal Disease Reporters (CADRs), presents a significant opportunity to investigate the effectiveness of rabies control measures. This ASAL is distinguished by low rainfall, dry terrain, and unfavorable environmental circumstances (13). The nomadic nature of a large segment of the county’s population involves regular migration with family members and livestock in quest of food, pasture, water, and shelter. These nomadic pastoralists are socioeconomically and politically underprivileged and lack access to modern facilities and basics such as education, healthcare, and transportation, among many other things (14). Due to frequent relocation, this community often has erratic access to up-to-date information, education, and medical services, including different intervention programs (15). The remote landscape and constant movement of pastoralist communities provide significant obstacles to large-scale canine vaccination campaigns, a difficulty also encountered in other regions of Kenya (16). As nomadic livestock keepers, their practices of drinking raw animal food like milk or blood, inadequate nutrition, and lack of access to medical facilities place these populations at higher risk of exposure to zoonotic diseases such as rabies, brucellosis, echinococcosis, bovine tuberculosis, Rift Valley fever and so on (17, 18).

In Turkana, rabies has long been a severe public health concern. The County One Health Unit (COHU) is undertaking numerous significant projects to address this issue. The County Veterinary Services, in collaboration with the Kenya Women Veterinary Association (KWVA), has undertaken several initiatives, such as the strategic vaccination and sterilization campaigns in sub-counties that pose a higher risk of rabies transmission. Dogs are not frequently brought to veterinary hospitals for care or treatment; instead, veterinary services are primarily provided on a mobile basis in Turkana. At the national level, priority is placed on post-exposure prophylaxis for humans rather than prophylactic vaccination for dogs. Therefore, to support the rabies control efforts of the Turkana County Veterinary Services team, this study aims to describe the knowledge, attitudes, and practices of the Turkana community people regarding dog rabies. We hypothesized that the Turkana community would possess satisfactory knowledge, attitudes, and practices concerning dog rabies. This study is the first to report basic information about rabies in dogs and the KAPs of people regarding rabies in Turkana County, Kenya.

## 2 Methods

### 2.1 Administrative structure of veterinary and animal health services in Turkana County, Kenya

**Figure 1:**
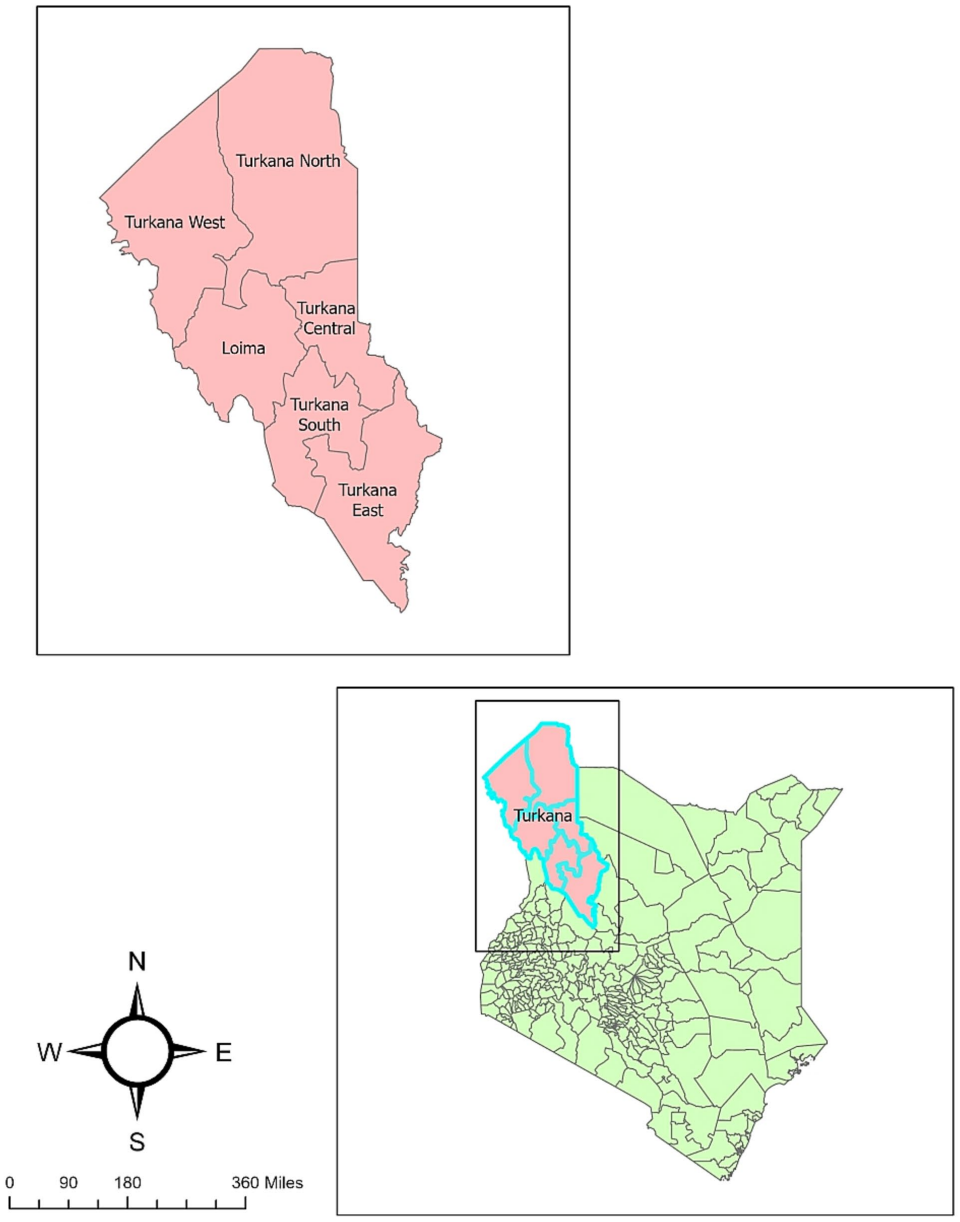
Map of Kenya highlighting sub-counties of Turkana County.

**Figure 2:**
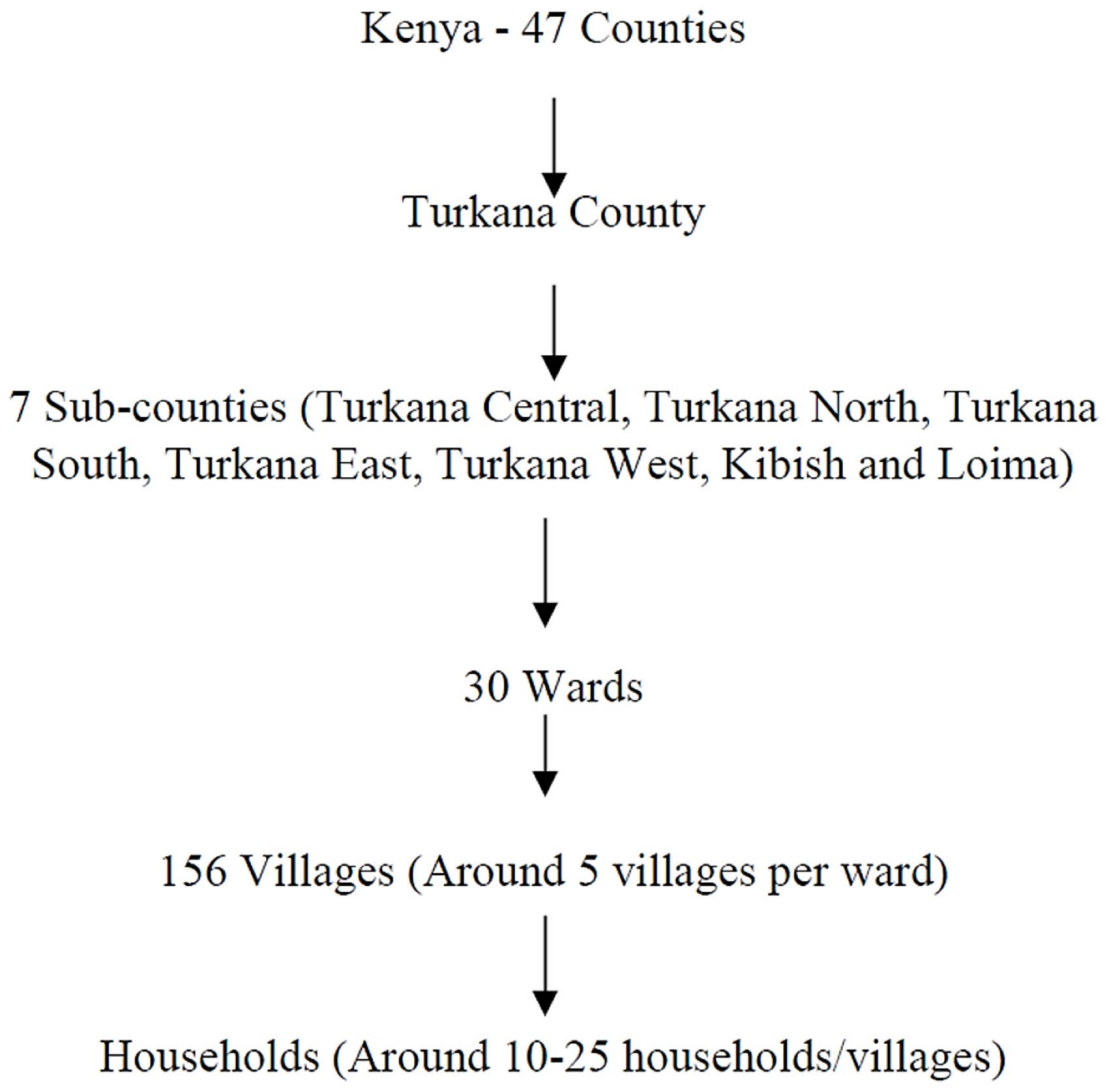
Administrative levels of Kenya.

**Figure 3:**
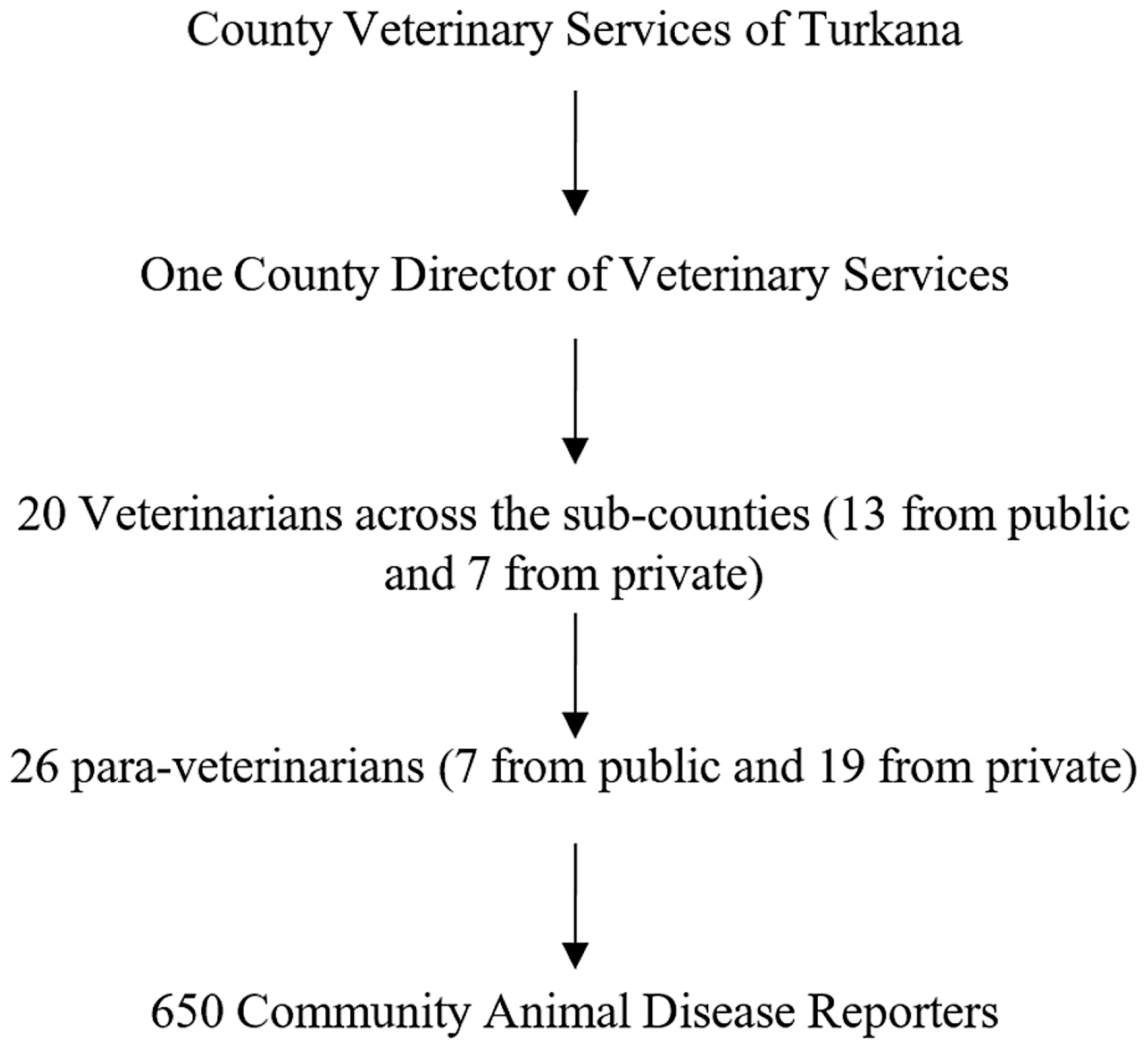
Hierarchy of veterinary professionals in Turkana.

**Figure 4:**
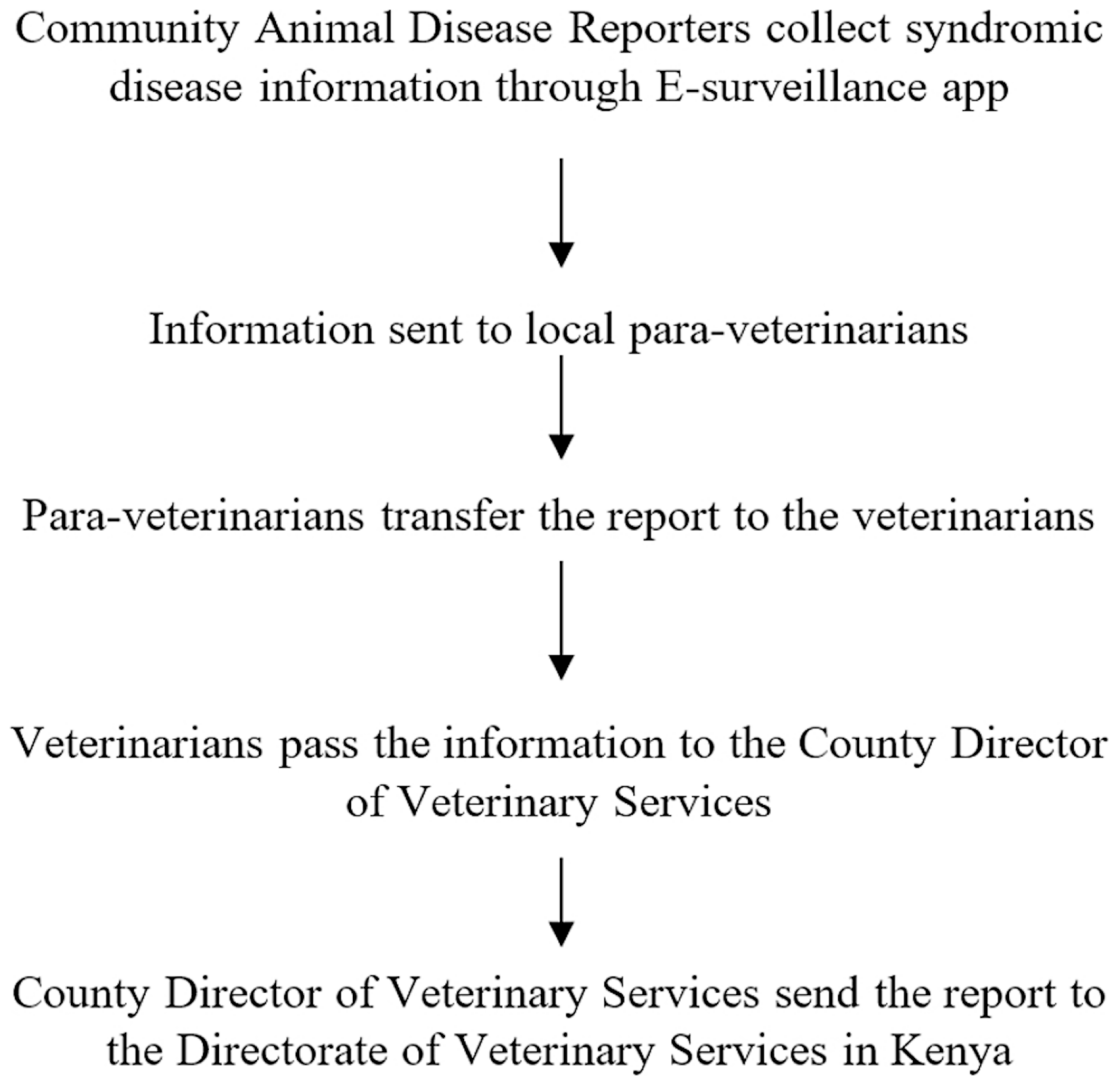
The flow of veterinary disease reporting from the peripheral to the central level of Kenya.

Turkana County is divided into seven sub-counties: Turkana Central, Turkana East, Turkana West, Turkana North, Turkana South, Loima, and Kibish (11) (Figure 1). The County Director of Veterinary Officer’s (CDVO) report indicates that these sub-counties consist of 30 wards, each including approximately five villages for a total 156 villages. Approximately 10–25 households are anticipated to be present in each village (Figure 2). In the County Veterinary Service of Turkana, one CDVO is appointed. In the seven sub-counties, there are 20 veterinarians, with 13 working in the public sector and seven in private practice. In addition, 26 para-veterinarians work in the region, with seven serving in public and 19 in private practice. So far, 650 CADRs are distributed in Turkana, with each village having between 2 to 6 CADRs present (Figure 3). The CADRs undergo a 21-day training program focused on fundamental animal health. They utilize an E-surveillance mobile application to gather and document disease data based on clinical signs. The data is initially assessed by local para-veterinarians, who pass it on to veterinarians. Veterinarians transmit the information to the CDVO, which passes it to the Directorate of Veterinary Services in Kenya (Figure 4).

### 2.2 Expert knowledge solicitation

To elucidate the present understanding and management of canine rabies, we surveyed a group of animal health workers regarding the KAPs (Knowledge, Attitudes, and Practices) of the people in their service areas. The participants were selected from seven sub-counties in Turkana County, Kenya, and were brought together for a Rabies Workshop in November 2023. Selection criteria for participants included having at least two years of professional experience working closely with local communities in veterinary services and rabies control. This cut-off ensured that each participant had substantial knowledge of local practices, challenges, and trends. Participants included veterinarians, para-veterinarians, and CADRs, with each group selected to represent their specific roles and expertise. Veterinarians and para-veterinarians were chosen for their formal training in animal disease management. At the same time, CADRs were included for their pivotal role as first-line disease reporters and their deep-rooted connections to the communities they serve. We chose expert knowledge elicitation over direct community surveys due to the logistical challenges of conducting comprehensive surveys in remote and expansive areas like Turkana. Experts embedded in the community were deemed best suited to provide timely and relevant insights. Their professional experience and community ties allow them to recognize subtle trends and issues that may not be apparent in broader community surveys. This approach was validated by ensuring diverse representation from all seven sub-counties and through the professional qualifications and experience of the selected participants.

### 2.3 Participants

During participant selection, at least two individuals were chosen from each sub-county, with additional participants selected from central Turkana due to its higher concentration of veterinarians compared to other regions. Of the 33 participants, 13 were from central Turkana, 17 represented six other sub-counties, and three did not specify their service locations. Their years of service ranged from 2 to 26 years.

### 2.4 Survey methodology

We visited Turkana, Kenya, in November 2023 to observe the real field scenario of rabies control activities in the county and to collect data for the current study. A cross-sectional survey was administered using the Qualtrics platform (Qualtrics, Provo, UT) during the “Rabies Prevention and Control” workshop held in Turkana from November 2nd to 3rd, 2023. We arranged the workshop in collaboration with the KWVA and Turkana County Government. The survey was conducted online. The questionnaire was designed to be user-friendly, allowing participants to complete it on their mobile phone with survey access provided during the workshop. Trained facilitators were available to assist participants with any technical or language-related issues, ensuring accurate and comprehensive data collection.

We designed the questionnaire after a thorough consultation with researchers at the University of Minnesota who had previous experience conducting similar surveys elsewhere (Supplementary File S1). The questionnaire was pretested among some participants and organizers and revised accordingly. The questionnaire was anonymous and semi-structured, with both open and closed-ended questions. The questionnaire was divided into four parts: demographic information and information regarding knowledge, attitudes, and community practices regarding dog rabies management. Respondents were instructed to answer questions about their geographic service area. The selection of questions was formulated to evaluate community members’ knowledge, attitudes, and practices based on an extensive literature review (19–23). The knowledge section contained general knowledge about rabies disease, its clinical signs, common reservoirs, responsible dog ownership, and dog population control methods. The questions in the attitude section capture attitudes toward dog vaccination and willingness to vaccinate the pets with or without payment. Additional questions in the practice part asked about the rate of vaccinations in recent campaigns, dog-roaming time per day, the primary purpose of dog keeping, food sources for dogs, and the reasons for not vaccinating dogs.

Following the educational segment of the workshop, we facilitated a group discussion with the study participants to gather their insights on rabies immunization for dogs. This interactive brainstorming session allowed participants to share their ideas about improving dog vaccination coverage. Additionally, we collected insights regarding vaccine challenges and shared additional information beyond the formal presentations as requested.

### 2.5 Statistical analysis

**Figure 5:**
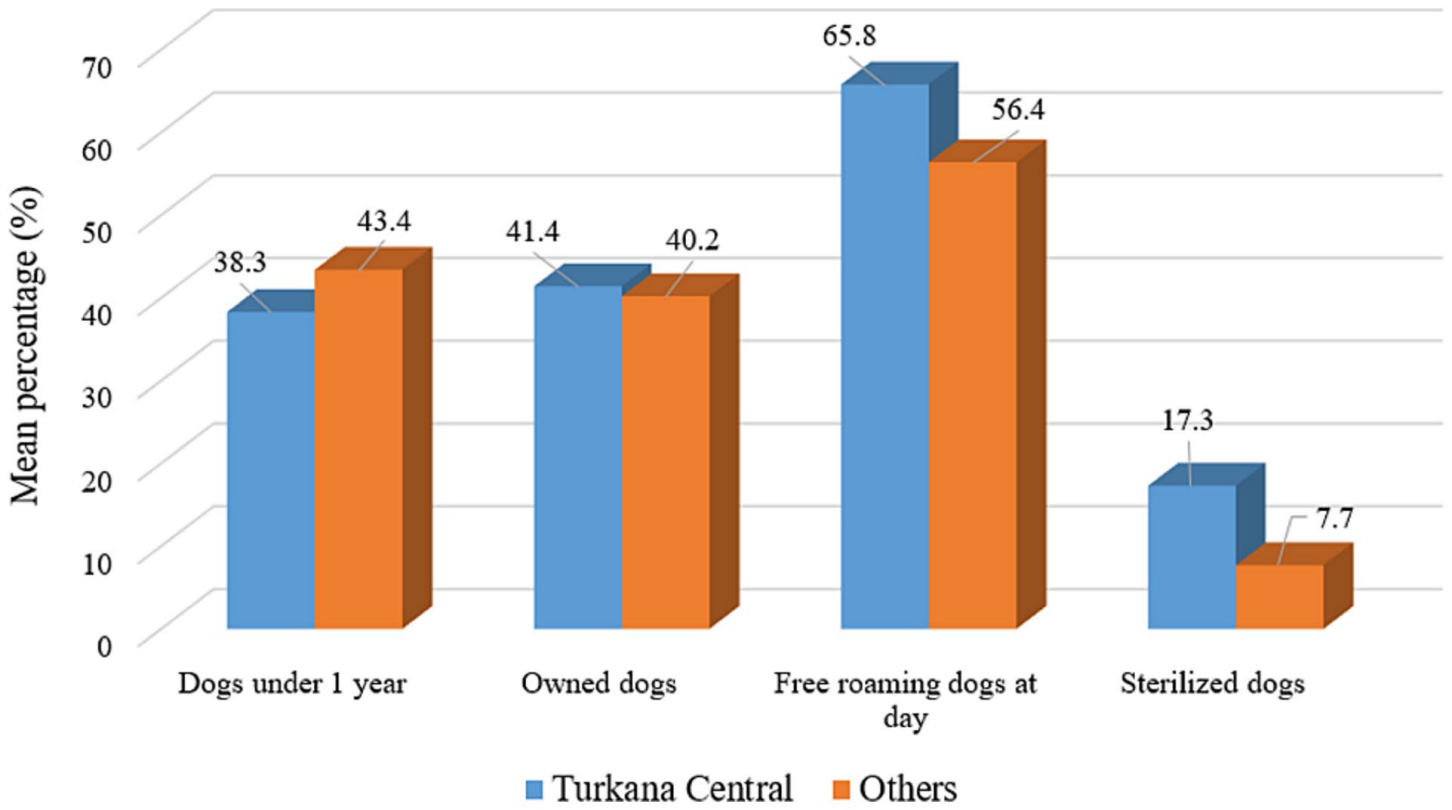
Demographic information on local dogs across locations in Turkana.

**Figure 6:**
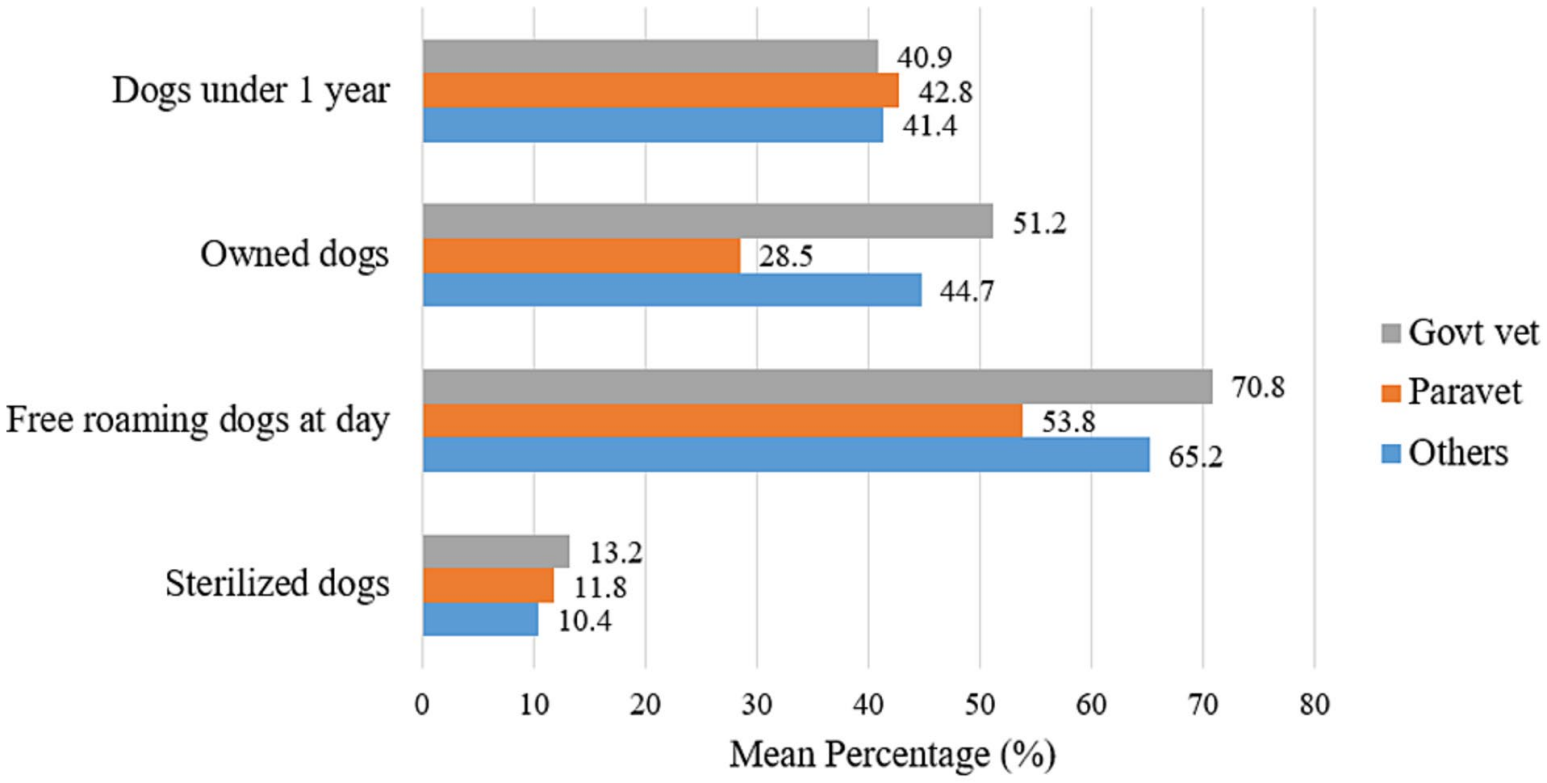
Demographic information on local dogs across different veterinary professions in Turkana.

**Figure 7:**
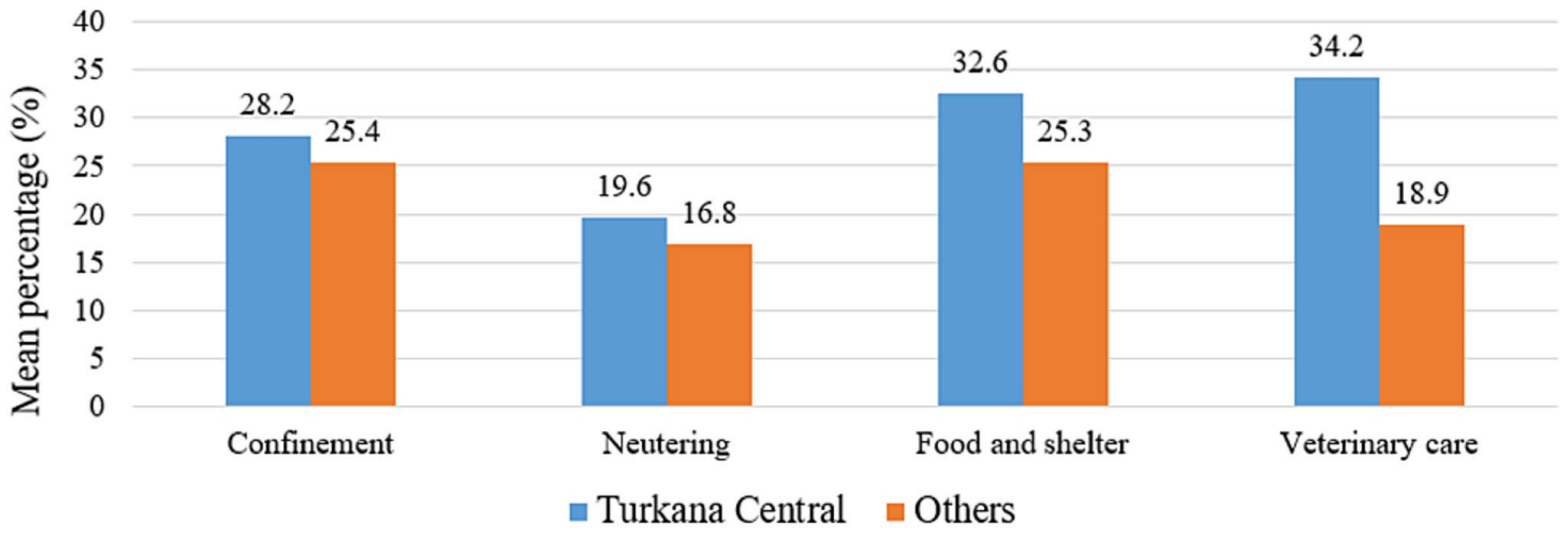
Estimated proportion of community members with positive attitudes toward different aspects of responsible dog ownership practices across locations in Turkana.

**Figure 8:**
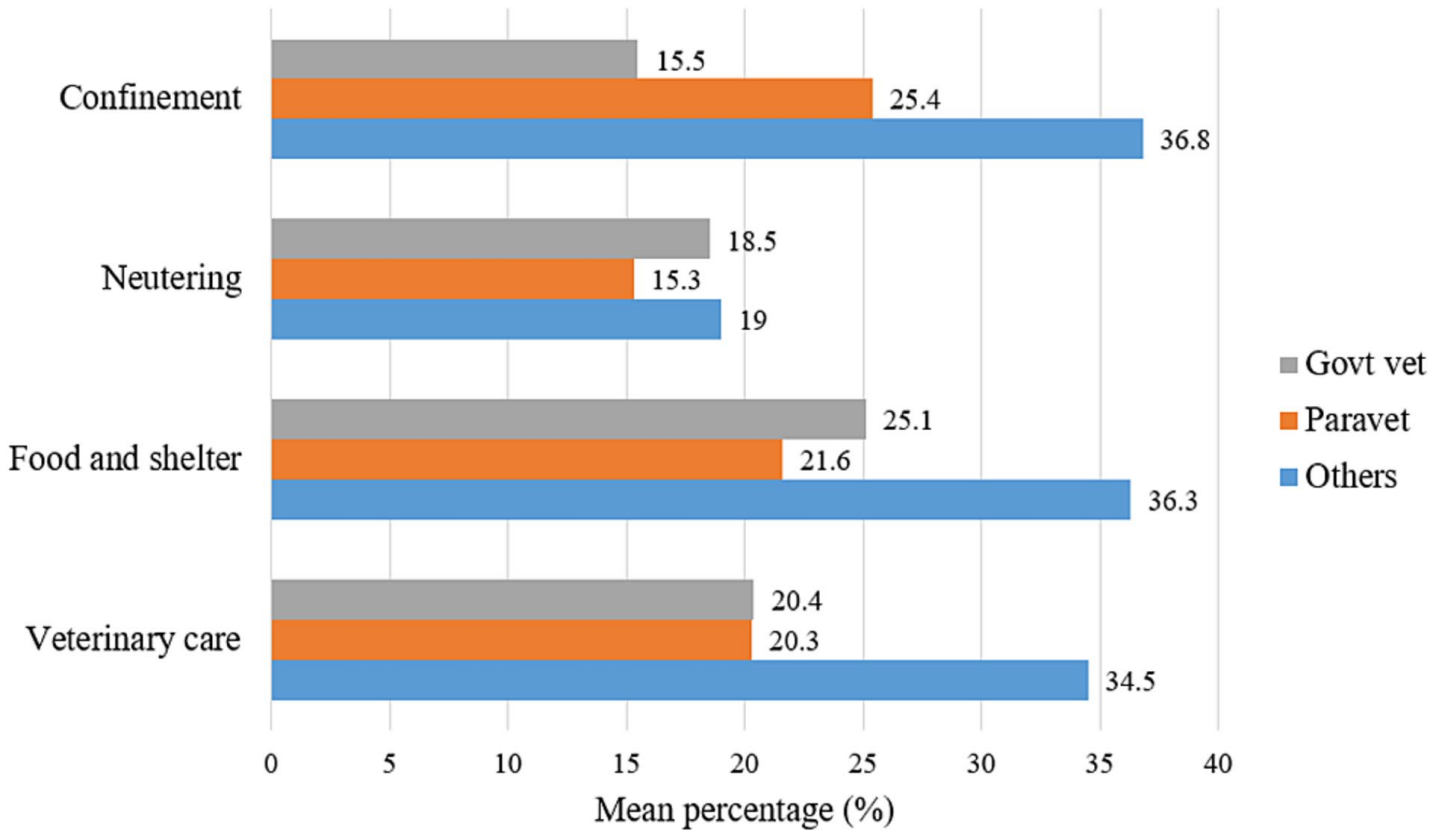
Estimated proportion of community members with positive attitudes toward different aspects of responsible dog ownership practices across different veterinary professions in Turkana.

Descriptive statistics for the categorical variables, such as frequency and percentages, were used to summarize the participant’s responses for each of the four survey sections (Table 1). Mean values were used for continuous variables (Figures 5–8). In the original questionnaire, the response items were categorized into four to five sub-categories, for example, 0–25%, 26–50%, 51–75%, 76–100%, and Not Sure. Based on the responses we obtained, we later collapsed most of the responses into two categories: below 50% and above 50%. We divided the original response categories into two groups because it simplifies the analysis and improves the results’ interpretability (24). The original categories had small sample sizes, which could lead to unreliable estimates and reduced statistical power. By combining categories, we aimed to achieve a more balanced distribution and enhance the robustness of our findings. We included the “Not sure” strata in the descriptive statistics but dropped this in further analysis due to minimal responses. Collapsing the sub-categories in certain variables was done due to small cell size. We broadly categorized the variable “current role” into three sub-categories: government veterinarians, para-veterinarians, and others (CADRs and private veterinarians) due to the small number of CADRs and private veterinarians in attendance. Participants from each of the seven sub-counties in Turkana participated, with the majority coming from Turkana Central. The location variable was categorized into three strata: Turkana Central, others, and “Unknown/Not mentioned.” Three participants from the third group did not mention their service location. Therefore, we excluded these three respondents from comparisons involving geographic location.

**Table 1:** Demographic information of the survey participants and summary statistics of the knowledge, attitude and practice related variables (*N* = 33).

Demographic information of the participants	Branches	Frequency (*n*)	Percentages (%)
Current role	Public veterinarians	12	36.4
	Para-veterinarians (Public and private)	12	36.4
	Others (CADRs and Private veterinarians)	9	27.3
Location (Sub-county)	Turkana Central	13	39.4
	Others (East, West, North, South, Kibish and Loima)	17	51.5
	Unknown/Not mentioned	3	9.1

Knowledge related variables	Expert opinion about community members	Frequency (*n*)	Percentages (%)
What percentage of people in your locality are aware of rabies?	Below 50%	14	42.4
	Above 50%	19	57.6
How do community members distinguish a suspected rabid animal?	By assessing the clinical signs of that dog	10	30.3
	After experiencing a dog bite or exposure event	13	39.4
	Both	10	30.3
What percentage of them can identify clinical signs of rabies in dogs?	Below 50%	25	75.8
	Above 50%	6	18.2
	Not Sure	2	6.1
What percentage of them are knowledgeable about dogs being a potential source of rabies for humans?	Below 50%	20	60.6
	Above 50%	13	39.4
What percentage of them know that rabies is fatal, once clinical signs appear in humans or animals?	Below 50%	21	63.6
	Above 50%	12	36.4
What percentage of people in your community know the significance of vaccinating dogs to prevent rabies transmission to humans and other animals?	Below 50%	28	84.9
	Above 50%	5	15.2

Attitude related variables	Expert opinion about community members	Frequency (*n*)	Percentages (%)
What percentage of people in the community exhibit a positive attitude toward vaccinating their dogs annually?	Below 50%	21	63.6
	Above 50%	12	36.4
In your opinion, what percentage of people are willing to vaccinate their dogs when it is free?	Below 50%	16	48.5
	Above 50%	17	51.5

Practice related variables	Expert opinion about community members	Frequency (*n*)	Percentages (%)
Approximately what percentage of dogs in your area were vaccinated during the most recent campaign?	Below 50%	29	87.9
	Above 50%	4	12.1
What is the common practice in your community regarding dogs being allowed to roam outdoors?	24 h	26	78.8
	Part of the day	6	18.2
	Not Sure	1	3.0
	Security and guarding	30	90.9
What are the primary purposes for which dogs are commonly kept in your area? (Check all that apply)	Hunting and Tracking	7	21.2
	Herding and Livestock Protection	21	63.6
	Companionship and Family Pets	14	42.4
	Cultural and Religious Significance	1	3.0
	Deterrence of Wildlife	9	27.3
	Provided by owners	3	9.1
How do the majority of dogs in your area typically source their food?	Scavenge food from public garbage	29	87.9
	Obtain food from restaurants	1	3.0

Differences in the proportions of participants depending on their locations were tested using either the Chi-square test or Fisher’s exact test (for cell size <5) (Table 2). According to participants’ responses, the differences were associated with the varying professions and were assessed with a total number of participants (33) (Table 3). In all these analyses, the cut-off value of the significance level was *p*-value ⩽ 0.05. The statistical differences in the means of several occupations were assessed using a one-way ANOVA (Analysis of Variance) test. In contrast, the categorical geographic location variables were tested using a two-sample *t*-test. The one-way ANOVA was used to compare the means of the dependent variables (e.g., average percentage of dogs under 1 year, average percentage of own dogs, average percentage of free roaming dogs and average percentage of sterilized dogs) across the three professional groups (Figures 6, 8). Likewise, the two-sample *t*-test was employed to compare the means of the dependent variables (e.g., confinement, neutering, food and shelter, and veterinary care) in both locations (Figures 5, 7). All these statistical analyses were done using an online calculator, “Social Science Statistics”.^1^ The map of Kenya highlighting Turkana was developed using ArcGIS Pro (Version 3.1.0) (ESRI Inc., Redlands, CA, United States).

**Table 2:** Knowledge, attitudes and practices related variables of the survey participants associated with different sub-counties of Turkana (*N* = 30).

Questions	Branches	Turkana central	Others	*p*-value
Knowledge related variables				
What percentage of people in your locality are aware of rabies?	Below 50%	5	8	0.43
	Above 50%	9	8	
How do community members distinguish a suspected rabid animal?	By assessing the clinical signs of that dog	5	4	0.73
	After experiencing a dog bite or exposure event	6	6	
	Both	3	6	
*What percentage of them can identify clinical signs of rabies in dogs?	Below 50%	12	11	1.00
	Above 50%	2	3	
What percentage of them are knowledgeable about dogs being a potential source of rabies for humans?	Below 50%	9	10	0.92
	Above 50%	5	6	
*What percentage of them know that rabies is fatal, once clinical signs appear in humans or animals?	Below 50%	9	10	0.89
	Above 50%	5	5	
What percentage of people in your community know the significance of vaccinating dogs to prevent rabies transmission to humans and other animals?	Below 50%	12	13	1.00
	Above 50%	2	3	
Attitude related variables				
What percentage of people in the community exhibit a positive attitude toward vaccinating their dogs annually?	Below 50%	7	11	0.30
	Above 50%	7	5	
In your opinion, what percentage of people are willing to vaccinate their dogs when it is free?	Below 50%	5	9	0.26
	Above 50%	9	7	
Practices related variables				
*Approximately what percentage of dogs in your area were vaccinated during the most recent campaign?	Below 50%	11	14	0.22
	Above 50%	2	0	
What is the common practice in your community regarding dogs being allowed to roam outdoors?	24 h	12	11	0.40
	Part of the day	2	5	

* We dropped the “Not sure” strata from the cross tab analysis.

**Table 3:** Knowledge, attitudes and practices related variables of the survey participants associated with different veterinary professions (*N* = 33).

Questions	Branches	Govt. vet	Para vet	Others	*p*-value
Knowledge related variables					
What percentage of people in your locality are aware of rabies?	Below 50%	6	5	3	0.90
	Above 50%	6	7	6	
How do community members distinguish a suspected rabid animal?	By assessing the clinical signs of that dog	4	1	5	0.20
	After experiencing a dog bite or exposure event	4	6	3	
	Both	4	5	1	
*What percentage of them can identify clinical signs of rabies in dogs?	Below 50%	9	8	8	0.96
	Above 50%	2	3	1	
What percentage of them are knowledgeable about dogs being a potential source of rabies for humans?	Below 50%	7	9	4	0.37
	Above 50%	5	3	5	
*What percentage of them know that rabies is fatal, once clinical signs appear in humans or animals?	Below 50%	9	6	6	0.54
	Above 50%	3	6	3	
What percentage of people in your community know the significance of vaccinating dogs to prevent rabies transmission to humans and other animals?	Below 50%	11	10	7	0.83
	Above 50%	1	2	2	
Attitude related variables					
What percentage of people in the community exhibit a positive attitude toward vaccinating their dogs annually?	Below 50%	10	8	3	0.06
	Above 50%	2	4	6	
In your opinion, what percentage of people are willing to vaccinate their dogs when it is free?	Below 50%	7	7	2	0.20
	Above 50%	5	5	7	
Practices related variables					
*Approximately what percentage of dogs in your area were vaccinated during the most recent campaign?	Below 50%	12	9	8	0.17
	Above 50%	0	3	1	
What is the common practice in your community regarding dogs being allowed to roam outdoors?	24 h	10	10	6	0.50
	Part of the day	2	1	3	

* We dropped the “Not sure” strata from the cross tab analysis.

## 3 Results

### 3.1 Demographic characteristics of the participants

Among 33 participants, 12 (36.4%) were Government veterinarians, 12 (36.4%) were para-veterinarians, and 9 (27.3%) were private veterinarians and/or CADRs. The region of Central Turkana accounted for 39.4% of the overall participants, while the remaining six sub-counties constituted 51.5% of the total respondents. Three respondents (9.1%) did not provide information on their service location in the survey (Table 1).

### 3.2 Community knowledge, attitudes, and practices about dog rabies

Out of the total sample size of 33 survey participants, 42.4% reported that less than half of their community members have knowledge of rabies. Most (57.6%) respondents expressed confidence that over half of their local population was aware of rabies. In relation to the identification of a suspected rabid animal, 39.4% of the participants indicated that people can identify a rabid dog after encountering a dog bite over half the time. Additionally, 30.3% of respondents mentioned that they can recognize a rabid dog by evaluating its clinical signs. The remaining 30.3% of participants reported both scenarios. According to 75.8% of participants, less than half of their community members had a good knowledge of the clinical signs of rabies in dogs, whereas only a smaller number (18.2%) replied that over half of their local people possessed that knowledge. Only 39.4% of participants claimed that the fact that dogs can act as a potential reservoir for rabies in humans is well understood by most people in their communities, whereas the remaining 60.6% mentioned that less than half of people had that knowledge. Most (63.6%) of the participants’ responses showed that only less than half of the community had awareness about the fatality of rabies. More than 80 % of participants thought that below 50% of their community members understood the importance of vaccinating dogs against dogs, while only 15.2% believed that more than 50% had that knowledge (Table 1). Based on the responses of 63.6% of the survey participants, it was found that a minority of individuals in Turkana held a favorable disposition toward ensuring annual immunization for their dogs. Besides, 51.5% of the participants indicated that more than half of the community members showed interest in getting the vaccine free. Regarding the administration of dog vaccinations in the recent campaign paid by the owners, nearly 90% of respondents said that the immunization rate is below 50%. In comparison, only 12.1% believed that the vaccination rate might be more than 50%. Furthermore, 78.8% of survey participants indicated that it is the standard practice in Turkana for community members to keep dogs exclusively outdoors. A smaller portion of the community, 18.2%, kept dogs outside for a part of the day and 3% were unsure about specific timing. The main reason for the people’s preference to maintain dogs is commonly attributed to security and guarding (90.9%), followed by herding and livestock protection (63.6%), companionship (42.4%), deterrence of wildlife (27.3%), and cultural and religious value (3%). Public garbage and leftovers were found to be the primary food source for the free-ranging dogs, accounting for 87–88% of their diet. Owners contributed some food (9.1%), while restaurants gave a little portion (3%).

### 3.3 Association between categorical variables

Tables 2, 3 display the associations of categorical variables related to knowledge, attitudes, and practices of dog rabies with the participant’s service locations (Turkana Central and others) and their varying professions (government veterinarians, para-veterinarians, and others). No association of variables with locations or professions was detected as statistically significant.

### 3.4 Association between continuous variables

Information about the dog population and responsible dog ownership practices of the dog owners were captured in the survey. In the sub-county of Turkana Central, on average, 38.3% of dogs were reportedly young (under one year of age), and 41.4% were “owned” dogs. The mean dog population roaming at some point in the day was recorded as 65.8%. Only 17.3% of dogs, on average, were reported as sterilized. Survey participants in the sub-counties outside of Central Turkana reported the mean number of young (43.4%) and owned dogs (40.2%), whereas the average number of free-roaming and sterilized dogs was reported as 56.6 and 7.7%, respectively (Figure 5). No statistically significant difference was detected.

We examined the same information on dog demography and responsible dog ownership practices of the dog owners reported by three professional categories: government veterinarians, para-veterinarians, and others (private veterinarians and CADRs) (Figure 6). Survey participants in the three professional categories reported very close opinions about the average number of young (40.9, 42.8, and 41.4%, respectively) and owned dogs (51.2, 28.5, and 44.7%) in their communities. The average number of free-roaming dogs was detected as 70.8, 53.8, and 65.2%, respectively, and sterilized dogs were recorded as 13.2, 11.8, and 10.4%, respectively. No statistical difference was found between responses by all three professional categories.

Positive attitudes toward different aspects of responsible dog ownership, such as confinement, neutering, providing food and shelter, and seeking veterinary care for dogs, were also recorded among various locations and respondent’s type of profession. While assessing Turkana Central and the other sub-counties, only veterinary care for dogs was found to be significantly associated (*p*-value = 0.03) among these four attitudes, with a response rate of 34.2 and 18.9%, respectively (Figure 7). In addition, while considering the three professional categories, responses were very close among all the four categories of responsible dog ownership. Private veterinarians and CADRs comparatively recorded the highest positive attitude rate (36.8%) toward the confinement of dogs than the public (15.5%) and the para-veterinarians (25.4%). Responses were very close for neutering dogs in the three professional categories, ranging from 15.3 to 19%. Similarly, concerning people’s attitudes about providing food, shelter, and medical care for pet dogs, the responses from the private veterinarians and CADRs were comparatively greater than those of the other two groups. However, no statistical differences in the means of these variables were detected (Figure 8).

After data collection and analysis, we conferred with some of the staff from the County Veterinary Services in Turkana to crosscheck the key findings in June of 2024. The validation process confirmed that these findings reflect the actual dog demographics and practices in Turkana. The staff from County Veterinary Services provided additional context that supported the lack of significant differences between regions and professional categories.

### 3.5 Barriers to dog vaccination

**Figure 9:**
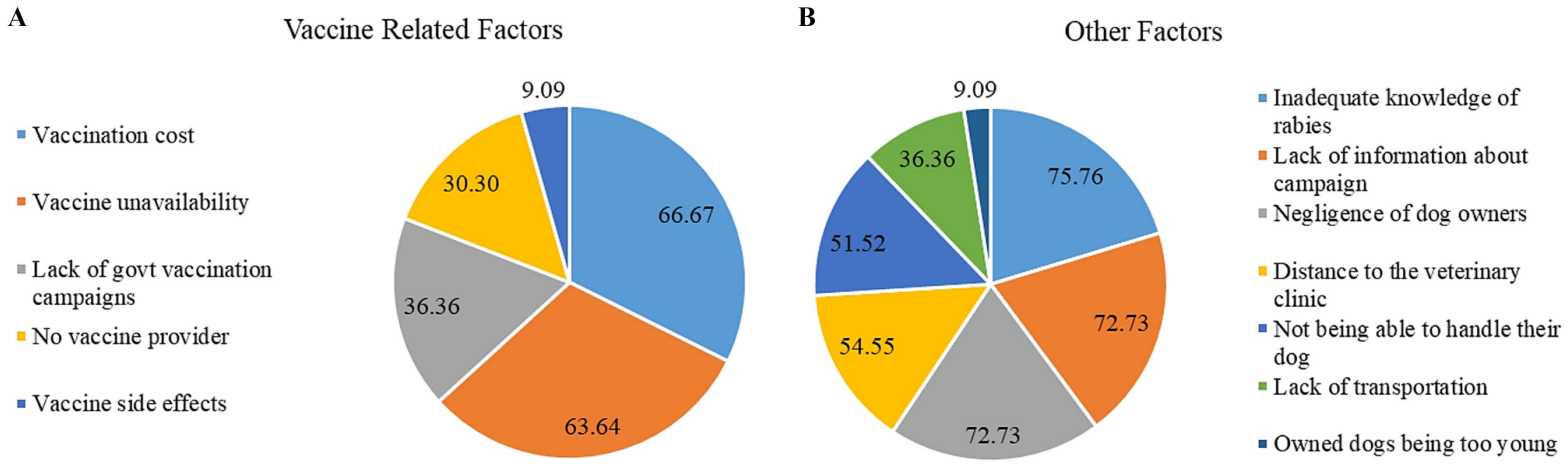
The barriers for dog rabies vaccination in Turkana; **(A)** vaccine-related factors and **(B)** other factors (percentages do not sum to 100% as multiple responses were allowed).

Our study participants identified several factors they believed to be contributing to the non-vaccination of dogs in their service areas: more than one response was allowed. We divided these factors into two categories: vaccine-related factors (Figure 9A) and other factors (Figure 9B). Vaccine-related factors include the high cost of vaccination (66.7%), unavailability of vaccines (63.6%), reduced government vaccination campaigns (36.4%), shortage of vaccine providers (30.3%), and concerns about side effects (9.1%). Other leading factors are owners’ insufficient knowledge about rabies (75.8%), lack of campaign information (72.7%), negligence of dog owners (72.7%), distance to clinic (54.6%), inability to handle/restrain dogs (51.5%), poor transportation (36.4%), effectivity of vaccine (9.1%), and very young age of dogs (9.1%). Additional factors contributing to reduced vaccination coverage of dogs in Turkana County surfaced during the structured discussion portion of the workshop. These factors include vaccination campaigns during school hours, the sheer high number of free-roaming dogs, and the lack of cold chain facilities. The post-workshop validation discussions further emphasized the critical barriers of vaccine cost, unavailability, and logistical challenges. County Veterinary staff noted that these barriers align with long-standing issues in Turkana, further supporting the study findings.

These findings highlight several critical areas for improving Turkana rabies control practices and policies. For instance, enhancing public awareness about rabies, its fatality, and the importance of dog vaccination can address knowledge gaps and increase participation in vaccination campaigns. Addressing logistical barriers such as vaccine unavailability, cold chain issues, and campaign timing could significantly improve vaccination coverage. Furthermore, promoting responsible dog ownership practices, including confinement and sterilization, is essential for reducing free-roaming dog populations, which play a pivotal role in sustaining rabies transmission. These targeted efforts, aligned with the identified barriers, can substantially strengthen the county’s rabies elimination strategy.

## 4 Discussion

In contrast to other research, our study categorized the responses into “above 50%” and “below 50%”. Although a single percentage statistic gives a broad understanding of awareness, our layered approach also provides significant detailed insights. According to the study results, most survey participants believed that over half of their local community members were aware of rabies. Many instances in previously conducted studies in the Philippines, Ethiopia, Azerbaijan, and Cambodia captured a high level of rabies awareness (> 90%) in their localities (25–28). In that case, it indicates that although general rabies awareness may be high, there may be a considerable community subgroup with little understanding of the disease. Besides, 42.4% of responses supported an awareness level below 50%, meaning a substantial portion of people in Turkana lack basic information about rabies.

Several studies demonstrated that while most individuals are aware of rabies, they possess a limited understanding of specific preventative measures and circumstances that increase the risk of contracting the disease (7, 29). In our study, approximately 30% of the individuals surveyed indicated that community members could identify a potential rabid animal by assessing the clinical symptoms, whereas 39.4% indicated such identification was possible after being bitten by the animal. The remaining 30% expressed their opinion that both methods were acceptable. Regarding clinical signs of rabies in dogs, around 76% of participants answered that less than 50% of the community members could identify the signs in a dog, and only 18% said it was more than 50%. The gap might be due to Turkana’s higher proportion of pastoral people and lower socio-economic status than other parts of Kenya (30). Due to frequent migration, this population poses unique challenges in accessing conventional health delivery systems (18). This finding is much lower than the other studies, where 84.4, 71, and 65.5% of study participants in Nigeria, Zimbabwe, and Burkina Faso could identify significant signs of a rabid dog (7, 29, 31). In Ethiopian pastoral communities, knowledge about rabies epidemiology, including its transmission cycle, reservoirs, and clinical signs in humans and dogs, is similarly limited (32). People believe that water scarcity, exposure to harsh winds, and the consumption of spoiled food could be potential causes of the disease in dogs (32). They often avoid seeking medical attention even after a dog bite exposure, and one of the main reasons for that is the limited awareness of the fatal consequences of rabies (32). In the current study, most survey respondents reported that less than half of their community members expressed concern regarding rabies’s zoonotic significance and lethality. Similar results were reported by other researchers, indicating that only a few individuals were aware of the deadly nature of rabies (7, 25, 29, 31, 33). Our survey revealed that most respondents (85%) believed that less than half of the Turkana population were aware of the usefulness of dog vaccination in preventing rabies, findings that align with the results of other studies conducted in Nigeria and Indonesia (9, 34, 35). All of this information suggests a significant requirement to educate dog owners and the general people regarding the hazards associated with rabies.

Mass dog vaccination is one of the most significant preventive measures for controlling rabies in dogs, thereby preventing human cases (36). According to the survey, less than half of the community responded positively to vaccinating dogs. However, they expressed a desire to vaccinate when the vaccines were provided free of charge. This finding has been replicated in several additional studies, where people expressed interest in pet vaccination when offered free of cost (20, 27, 28, 37, 38). Following the statement of CDVO of Turkana, to sensitize the people to dog vaccination, private veterinarians are provided with various forms of government support, such as motorcycles and petrol for transportation to patient appointments. However, despite having these resources, no designated place is available to organize a campaign. In addition, rabies vaccines for dogs are considerably costly in private clinics in Kenya, ranging from 1,000 to 2000 Kenyan shillings or approximately 7 to 15 USD (39). Conversely, these vaccines are free of charge for dog owners during mass dog vaccination campaigns sponsored by the Government and different partner organizations (Source – KWVA). It indicates a demand for more such campaigns in Turkana, indicating the necessity of expanding immunization efforts to cover a larger county area. It also underscores the requirement of raising awareness among dog owners regarding the significance of rabies vaccination to enhance their involvement in these campaigns.

These reasons reflect the practice of community people regarding vaccinating their dogs in recent campaigns. Around 88% of survey respondents implied that less than half of their community people participated in the recent vaccination campaign, meaning that many dog owners did not vaccinate their animals. This result supports the previous studies conducted in different parts of Africa that revealed the percentage of dog vaccination in particular areas below 25% (31, 40–43). Our study demonstrated that owners prefer to permit their dogs to roam outside 24 h a day (78.8%) rather than restricting them to a particular time in the yard or house. These findings correspond with another study in Kenya, which reported that 68.6% of dogs were allowed to wander freely in Kisumu County (44). Most houses in Turkana have no fences, while some have low-height bamboo fences. However, some houses in urban settings have brick dog kennels. This scenario could be attributed to the fact that in rabies-endemic areas of Kenya, some dog owners may provide minimal care for their pets, including limited access to food, medical attention, and movement restrictions (44). Millions of pastoralists worldwide rely on livestock rearing for their livelihoods and keep dogs for herding and protection. This mobile community, along with their livestock and pets, often faces malnutrition, creating conditions that foster the spread of various infectious diseases (45). Likewise, in Turkana, the primary motivations for keeping dogs were security and guarding, followed by herding, livestock protection, and companionship. Additional factors include protection from wildlife, hunting, and religious convictions. Many countries worldwide commonly keep dogs for security and to safeguard their owners (31, 43, 46–48).

Our survey respondents identified several factors contributing to dog owners’ decision not to vaccinate their dogs against rabies. Regarding vaccine-related factors, the cost of vaccines emerged as the most significant reason for the lack of vaccination in dogs, which aligns with other African studies (9, 20, 23, 49). In Kenya, private practitioners often charge substantial fees for rabies vaccines. Conversely, while mass dog vaccination campaigns organized by the government and other partners are free of charge, these campaigns are infrequent. Another reason for the high cost could be the shortage of cold-chain facilities in non-central sub-counties. These facilities are only available at the headquarters in Lodwar, which is far from other areas, leading to significant transportation costs. The unavailability of rabies vaccine (63.6%) was another leading cause of immunization in Turkana, which is consistent with the findings of Dahourou et al. (23). The absence of government vaccination programs (36.4%), the unavailability of vaccine providers (30.3%), and worries over vaccine side effects (9.1%) are other vaccine-related factors that contribute to the insufficient dog vaccination rates. A study conducted in Uganda revealed that the lack of government-led vaccination efforts was responsible for 18.5% of the reasons cited for not being vaccinated. This percentage is approximately half of the figure we obtained (23). Another study from Tanzania found that only 1 % of the 750 households surveyed expressed concern regarding the vaccine’s adverse effects (46). In other factors, the primary reason has been unveiled as an insufficient understanding of rabies (75.8%). It could be attributed to rabies control initiatives prioritizing vaccination and sterilization rather than implementing a holistic strategy to raise awareness among the population. Sensitization occurs exclusively during campaigns conducted by focal persons or administrators. Additional prominent factors include inadequate awareness of the vaccination campaign (72.7%) and owners’ disinterest in vaccinating their pets (72.7%). Some dog owners in specific regions neglect to vaccinate their pets due to uneducated ownership practices. Some do so without any legitimate reasoning, while others hold the belief that vaccines are either ineffective or potentially harmful to their dogs (20, 23, 50, 51).

Immunization campaigns during school hours were found to be a common cause for not vaccinating the dogs in Turkana. It is typical in Turkana for nearly every child in a family to own a pet dog. Consequently, campaigns organized during school hours resulted in many dogs remaining unvaccinated as the children could not take the dogs to the campaign site. The immunization campaign can be scheduled after school hours or on weekends to prevent such circumstances. Free-roaming dogs refer to dogs without specific owners who have unrestricted access to public stuff (52). These ownerless dogs mostly have little chance of being vaccinated during a campaign. Participants reported the essential preliminary measure is to enhance community awareness of the significance of rabies vaccination. It includes educating the community about rabies as a human health threat, administering rabies vaccines to dogs, and motivating them to pursue vaccination. The absence of cold chain infrastructure in Turkana has been identified as another leading factor and shortcoming in dog vaccination. This finding aligns with studies previously conducted in Kenya (53) and India (54). In Kenya, more than 50% of the sub-counties have dedicated refrigerators at the sub-county level for storing routine post-exposure human rabies vaccines. However, there is a shortage of dedicated refrigerated units for non-routine vaccines. This scenario could be the same as the case of dog rabies vaccines at the periphery levels of the country.

Our study found that the average proportion of dogs under one-year-old is 38.3% in Turkana Central and 43.4% in other parts; this is slightly lower than an overall estimate of 52% reported in a survey performed in Tanzania (55). According to the County Deputy Director of Veterinary Service in Turkana, this might be due to the limited sterilization campaigns, primarily concentrated in the headquarters in Turkana Central. The substantial growth of this dog population requires improved mobilization of resources, such as increased efforts in vaccination and deworming, as well as ensuring an adequate supply of medication. The rapid population rise may lead to a corresponding spike in stray or undesirable puppies. Consequently, it emphasizes the need for efficient population control measures, such as neutering or spaying campaigns, to manage the growing dog population. According to the survey results, around 60% of dogs in the county are ownerless, and 66% roam at some point during the day. In discussion with our Turkana partners, we learned that most dogs are closely attached to somebody in the community but may not be considered “owned” in the Western sense of the word, and the dogs may or may not be fed or otherwise cared for. Understanding this nuance is essential because young and free-roaming dogs constitute a substantial portion of the dog population, and their inclusion in vaccination campaigns is necessary to achieve comprehensive coverage and successful disease control. Failure to vaccinate many young dogs creates an ongoing population of vulnerable animals, which can sustain the rabies virus circulation (56). The study also revealed that only 17.3% of the typical dog population in Turkana Central and 7.7% in the rest of the sub-counties undergoes sterilization, which is closer to the findings from Indonesia (12%) (50) and the Philippines (13%) (57). This sterilization rate is more than double in Central Turkana than in other parts because of the limited resources available in the outside sub-counties rather than the headquarters. The shortage of human expertise, i.e., veterinarians and para-veterinarians, is mainly because they are hired from the neighboring sub-counties to staff the spot campaigns. Another reason for the higher sterilization rate in the Central part might be the more significant number of private practitioners in the urban settings who frequently carry out sterilizations than in the other parts. Turkana exhibits less than 35% positive attitudes toward responsible dog ownership practices, such as confinement, sterilization, and ensuring canines are provided with food, shelter, and medical attention. Out of all, only providing veterinary care to dogs was found significantly higher in Turkana Central compared to other areas where this is a major challenge. This is due to the greater accessibility of public and private veterinarians, as well as clinics, in the Central town. Lower attitude level also reflects the insufficient education regarding responsible dog ownership and population management. Raising awareness and involving the community in advocating for ethical dog ownership can promote the behavioral changes required for effective rabies management (58). Turkana does not have a well-organized government veterinary hospital, although there are a few private practitioners and clinics. Mobile services largely tend to food animals and are hardly offered for dogs and cats.

A secondary objective of this study was to evaluate the utilization of various categories of veterinary professionals to represent their communities of service as “experts.” Our results indicate relatively uniform survey responses from all participants regardless of their professional role in rabies control. This finding suggests that utilizing any professionals can contribute valid surveillance data for rabies control efforts in Turkana. This finding is helpful as the number of CADRs far exceeds the number of veterinarians and para-veterinarians in Turkana County (Figure 3), and the service area of CADRs is comparatively much smaller compared to veterinarians and para-veterinarians. CADRs are selected based on their leadership skills in the community. They are mapped and can be located through GPS. Some counties, including Turkana, have CADRs that are supported by USAID and managed by the FAO. CADRs utilize a cell phone-based disease reporting system. This reporting system could be modified to collect detailed information for dog population estimates, vaccine supply needs, and vaccination campaign coverage. CADRs may also be able to tailor vaccine campaign sensitization to be more specific and practical within their communities.

Achieving the sustainable elimination of rabies dictates a combination of community involvement, education, robust vaccination initiatives, and enhanced infrastructure to deliver enduring solutions. Investigating community-driven initiatives for rabies control and canine population management may yield significant information. It may include pilot initiatives that enable local communities to oversee dog populations and educate their counterparts. One concrete step could involve leveraging existing community leadership, such as CADRs, to organize targeted awareness campaigns about rabies prevention and vaccination benefits. Additionally, collaboration with schools and local leaders to integrate rabies education into community programs could ensure that knowledge reaches both children and adults. These efforts could significantly improve understanding and participation in vaccination campaigns. An integrated approach could be highly effective in this context. Rabies vaccines for dogs could be administered during routine livestock vaccination programs. Animal health workers visiting households could also provide rabies vaccinations, administer deworming treatments, and fit dogs with parasite-repellent collars (45). Future research should focus on the long-term effects of mass vaccination initiatives on rabies prevalence in human and animal populations.

## 5 Limitations of the study

As in all cross-sectional surveys, there are several places where bias could be introduced into our study. The small sample size limits the statistical power of our findings and may obscure significant differences that could emerge with a larger sample. The participants were selected based on their professional roles and experience, so their responses may reflect their perspectives and experiences, which may not fully represent the broader community’s views. A diverse group of experts from various sub-counties was included to help mitigate the potential bias to address this limitation. The reliance on self-reported data introduces potential biases, such as response or recall bias, as participants may provide socially desirable answers or fail to recall information accurately. Additionally, classification bias may have occurred if participants felt pressured to provide specific responses. While these limitations are inherent in self-reported data, they are unlikely to significantly affect the findings, as they align with trends observed in similar studies. The findings from our research in Turkana are likely applicable to other regions with similar socio-economic and cultural settings in Kenya. Areas with comparable challenges, such as remote locations, limited veterinary resources, and similar livestock management practices, can benefit from the insights and recommendations derived from our research.

## 6 Conclusion

The current study differs from other traditional KAP-related studies because the data were not collected directly from the community members but from a group of expert veterinary persons in the field. The study participants shared their knowledge regarding rabies-related questions based on their professional experience. The research findings indicate that the rabies-related knowledge, attitudes, and practices among the Turkana community are unsatisfactory across all sub-counties. Notably, the consistency in data from different veterinary professionals ensures the reliability of the sources, regardless of their specific roles, and represents a cost-effective data collection method. The report also emphasizes the substantial population of young dogs in Turkana County, highlighting the need for strict planning for vaccination allocation and humane population management. It highlights the importance of raising awareness within the community about the sources of rabies, how it spreads, the risks it poses to humans, the prompt reporting of bite incidents, and responsible ownership of dogs, including vaccination and sterilization. Ultimately, the results of this study will offer a reliable foundation of information for policymakers regarding the community’s understanding of dog rabies and how to plan for the following steps to control rabies in Turkana County, Kenya.

## Data Availability

The raw data supporting the conclusions of this article will be made available by the authors, without undue reservation.

## Footnotes

^1^ https://www.socscistatistics.com/tests/
