# Supplementary material for: Assessing veterinary professionals’ perspectives on community knowledge, attitudes, and practices regarding dog rabies in Turkana, Kenya
Source: Front Vet Sci. 2025 Mar 13;12:1526551. doi: 10.3389/fvets.2025.1526551 (PMC11948536; doi:10.3389/fvets.2025.1526551)
Supplement: Supplementary file 1 [file Data_Sheet_1.PDF]

Supplementary file (S1 File): Survey questionnaire

Demographic information

Q1: What is your current role?

- Government veterinarian
- Para-veterinarian (Public and private)
- Community Animal Disease Reporters (CADR)
- Private veterinarian

Q2: What is your current affiliation?

Q3: How many years of public/private practice do you have?

Q4: Please mention the location of your place of work (Sub-county, ward)

Q5: In your opinion, in your area what percentage of the dog population is under 1 year old?

Q6: In your opinion, in your area - - What percentage of the dog population is owned?

Q7: In your opinion, in your area - - What percentage of the dog population are free roaming at some point during the day?

Q8: In your opinion, in your area - - What percentage of the dog population is sterilized?

Q9: What is the average number of dogs per household in your area?

Knowledge related questions

Q10: What percentage of people in your locality are aware of rabies?

- Below 50%
- Above 50%

Q11: Do you estimate the number of dogs in your area? If so, then how?

Q12: Over the last 5 years, is the dog population in your area-

- Increasing
- Decreasing
- Not sure

Q13: How do community members distinguish a suspected rabid animal?

- By assessing the clinical signs of that dog
- After experiencing a dog bite or exposure event
- Both

Q14: What percentage of them can identify clinical signs of rabies in dogs?

- Below 50%
- Above 50%
- Not sure

Q15: What percentage of them are knowledgeable about dogs being a potential source of rabies for humans?

- Below 50%
- Above 50%

Q16: What percentage of them know that rabies is fatal, once clinical signs appear in humans or animals?

- Below 50%
- Above 50%

Q17: What percentage of people in your community know the significance of vaccinating dogs to prevent rabies transmission to humans and other animals?

- Below 50%
- Above 50%
- Not sure

Q18: Approximately, what percentage of people in your community are aware of the appropriate rabies vaccine schedule in dogs (annual vaccination)?

- Below 50%
- Above 50%
- Not sure

Q19: What percentage of people in your community know that the leashing and confinement of dogs are integral aspects of responsible dog ownership?

- Below 50%
- Above 50%

Q20: What percentage of people know about dog population control methods (neutering/ sterilization)?

- Below 50%
- Above 50%

Attitude related questions

Q21: What percentage of people in the community exhibit a positive attitude toward vaccinating their dogs annually?

- Below 50%
- Above 50%

Q22: In your opinion, what percentage of people are willing to vaccinate their dogs when it is free?

- Below 50%
- Above 50%
- Not sure

Q23: In your opinion, what percentage of people would be willing to vaccinate their dogs by paying the cost (such as, co-pay)?

- Below 50%
- Above 50%
- Not sure

Q24: What percentage of people in the community demonstrate a positive attitude toward responsible dog ownership practices, including confinement?

Q25: What percentage of people in the community demonstrate a positive attitude toward responsible dog ownership practices, including neutering/sterilization?

Q26: What percentage of people in the community demonstrate a positive attitude toward responsible dog ownership practices, including providing food and shelter?

Q27: What percentage of people in the community demonstrate a positive attitude toward responsible dog ownership practices, including seeking veterinary care?

Practice related questions

Q28: Approximately what percentage of dogs in your area were vaccinated during the most recent campaign?

- Below 50%
- Above 50%
- Not sure

Q29: What is the common practice in your community regarding dogs being allowed to roam outdoors?

- 24 hours
- Part of the day
- Not sure

Q30: What are the primary purposes for which dogs are commonly kept in your area? Check all that apply.

- Security and guarding
- Hunting and Tracking
- Herding and Livestock Protection
- Companionship and Family Pets
- Cultural and Religious Significance
- Deterrence of Wildlife

Q31: How do the majority of dogs in your area typically source their food?

- Provided by owners
- Scavenge food from public garbage
- Obtain food from restaurants

Q32: Why do dog owners in your area not vaccinate their dogs against rabies? (Check all that apply)

- Vaccination cost
- Unavailability of the vaccine
- Lack of government vaccination campaigns
- No vaccine provider
- Concerns about vaccine side effects
- Inadequate knowledge of rabies
- Lack of information about the campaign
- Negligence of dog owners/A belief that dog vaccination is useless
- Distance to the veterinary clinic
- Not being able to handle their dog
- Lack of means of transportation
- Owned dogs being too young
